# Assessing the Safety and Efficacy of Rivaroxaban for Stroke Prevention in Patients With Atrial Fibrillation: A Systemic Review and Meta-Analysis

**DOI:** 10.7759/cureus.54252

**Published:** 2024-02-15

**Authors:** Ghazala S Virk, Sana Javed, Raheel Chaudhry, Mustafa M Moazam, Arhum Mahmood, Faraz Mahmood, Mohammed Zaheer, Shahroz M Khan, Vedika Rajasekaran

**Affiliations:** 1 Internal Medicine, Avalon University School of Medicine, Ohio, USA; 2 Medicine, University of Birmingham, Royal College of General Practitioners, Birmingham, GBR; 3 Medicine, Baylor College of Medicine, Houston, USA; 4 Psychiatry, Texas Tech University Health Sciences Center El Paso, El Paso, USA; 5 Internal Medicine, Henry Ford Health System, Detroit, USA; 6 Internal Medicine, Detroit Medical Center/Wayne State University School of Medicine, Detroit, USA; 7 Internal Medicine, Deccan College of Medical Sciences, Hyderabad, IND; 8 Medicine, Kansas Health Science Center (KHSC) College of Osteopathic Medicine, Wichita, USA; 9 Internal Medicine, WellStar Hospital System, Marietta, USA

**Keywords:** direct-acting oral anticoagulants (doacs), prevention of ischemic stroke, myocardia infarction, oral ixa inhibitors, thromboembolic events, atrial fibrillation (af), rivaroxaban, oral anticoagulation, embolism, stroke

## Abstract

An effective anticoagulation therapy is required for patients with atrial fibrillation because it presents a significant risk of stroke. The current study evaluates the relative safety as well as efficacy of rivaroxaban in patients who are diagnosed with atrial fibrillation. A thorough literature review of relevant databases was conducted, focusing on academic and clinical studies that were published from 2017 onward. Inclusion criteria comprised randomized controlled trials and other observational studies comparing the incidence of stroke and the safety index of rivaroxaban in atrial fibrillation. We followed the Preferred Reporting Items for Systematic Review and Meta-Analyses (PRISMA) for data overview reporting and overview. A total of 21 studies were selected based on the inclusion criteria. A total of 19/21 studies advocated the adoption of rivaroxaban for minimizing stroke incidence. Rivaroxaban also showed superiority in achieving the therapeutic objectives, i.e., reduction in the incidence of stroke. The results for rivaroxaban against warfarin showed an improved safety index and effectiveness of rivaroxaban. The total effect size for the analysis was calculated to be Z=2.62 (p-value=0.009). The individual effect of all studies favored the “rivaroxaban” group. The heterogeneity in the study was as follows: tau^2^=0.10; chi^2^=110.10, df=6; I^2^=95%. The second analysis for risk reduction and incidence of stroke after rivaroxaban therapy also showed a bias towards rivaroxaban therapy. The combined effect for the analysis was found to be as follows: HR=0.73 ((95% CI: 0.50, 1.07). The total effect was calculated to be Z=1.61 (p-value= 0.10). The heterogeneity was found to be as follows: tau^2^= 0.20, chi^2^=89.97, df=6, I^2^=93%. Standard dosing of rivaroxaban emerges as a preferred strategy for stroke prevention, balancing efficacy and safety. Clinical decision-making should consider individual patient characteristics and future research should delve into specific subpopulations and long-term outcomes to further refine treatment guidelines.

## Introduction and background

Atrial fibrillation (AF), characterized by irregular heartbeats, remains a serious health concern for the public because it correlates with the risk of embolism and stroke [1]. According to a recent report, approximately five million individuals in the USA are affected by AF. An enormous increase in these numbers (as many as 12 million) is expected within the next two decades [2]. AF is frequently treated with pharmacological therapies that mainly consist of direct oral anticoagulants. Within this drug group, rivaroxaban (a factor Xa inhibitor) has emerged demonstrating efficacy in stroke prevention and providing a further benefit of convenient dosing regimens [3]. Recent advancements in pharmacological strategies have achieved considerable therapeutic objectives [4]. Moreover, the comparative effectiveness of rivaroxaban with other anticoagulation therapies, such as Vitamin K antagonists and warfarin, is a subject of concern for clinicians and medical professionals. Recent trials demonstrate the comparative risks and benefits of rivaroxaban in comparison with conventional forms of pharmacological therapies [5].

Rationale

Timely intervention and adequate anticoagulant treatment measures for the prevention of stroke can provide early symptomatic relief and secure patient prognosis, ensuring improved clinical outcomes. Large-scale trials and observational studies have repeatedly advocated direct oral anticoagulants as a prominent treatment option based on their safety [6]. It is important to determine different characteristics of regimens of rivaroxaban through evidence-based studies and clinically oriented guidelines for the effective development of treatment strategies that cater to the specific needs and requirements of the patient with AF. Understanding the comparative effectiveness is crucial for tailoring anticoagulation therapy to individual patient needs, optimizing adherence, and potentially improving clinical outcomes.

Objectives

The current investigation aimed (i) to evaluate the efficacy of rivaroxaban in preventing stroke and other thromboembolic events in patients of AF, (ii) to explore variations in thromboembolic events, including ischemic strokes in long-term patient prognosis, (iii) to evaluate and compare the safety profiles of rivaroxaban in patients with AF, and (iv) to conduct a thorough assessment of publication bias by analyzing and reporting on potential selective reporting of outcomes in the included studies.

## Review

Definitions

AF is a cardiac arrhythmia wherein fast and disorganized electrical activation within the heart's atrial chambers leads to an irregular and often accelerated atrial rhythm. Consequently, blood cannot be adequately pumped into the ventricles. It is caused by abnormal impulse initiation and propagation from the sinoatrial node to the atrioventricular node. Stroke prevention involves measures to lessen the risk of thromboembolic events, particularly ischemic strokes. It encompasses anticoagulant therapy to mitigate the formation of blood clots in the atria [7-8]. Efficacy of rivaroxaban refers to its ability to achieve its intended therapeutic effect, while safety encompasses the assessment of negative events associated with the use of rivaroxaban, including significant bleeding incidents and clinically significant non-major bleeding and overall bleeding-related complications [9].

AF is distinct from other arrhythmias and features an irregular rhythm and specific electrophysiological features. It involves rapid, bumbling atrial activation due to ectopic discharges, usually originating from the pulmonary veins, leading to complex microcircuits in the atria. This precise focus on its unique electrical disturbances sets AF apart from other arrhythmias with similar irregular heartbeat symptoms.

Methods

Eligibility Criteria

We set the eligibility criteria according to the “Population, Intervention, Comparison, Outcome, and Study Design (PICOS)” scheme, as recommended by Preferred Reporting Items for Systematic Review and Meta-Analyses (PRISMA) guidelines. The inclusion criteria were as follows: (1) literature that was published from 2017-2023, (2) adults who had a confirmed diagnosis of AF, (3) studies investigating various dose-related therapeutic impacts of rivaroxaban, (4) studies comparing rivaroxaban with other anticoagulants, and (5) studies reporting efficacy in stroke prevention and safety outcomes (bleeding events). The exclusion criteria were as follows: (1) any study published before 2017, (2) non-observational studies and other review studies, (3) studies with a target population of diagnoses other than AF, and (4) studies that included the young pediatric population (Table 1).

**Table 1:** Inclusion and exclusion criteria for the review

Criteria	Inclusion	Exclusion
Language	English	All other languages
Timeframe of publications	2017–2023	Older
Type of studies	Qualitative, quantitative, mixed methods, case study	Prospective, protocols, reviews, grey literature
Region	All	-
Target population	Adults who had a confirmed diagnosis of atrial fibrillation; populations who are at low risk or have an underlying cardiovascular abnormality	Populations with health abnormalities other than cardiovascular disorders; studies with a target population of diagnoses other than AF
Context	Studies investigating various dose-related therapeutic impacts of rivaroxaban; studies comparing rivaroxaban with other anticoagulants	Studies including the young pediatric population
Type of health issue	Studies reporting efficacy in stroke prevention and safety outcomes (bleeding events)	Studies discussing other health-related outcomes

Information Sources

A number of digital databases were explored to retrieve relevant studies. These consist of ClinicalTrials.gov, PubMed, Google Scholar, ScienceDirect, Medline, Embase, Cochrane Library, and PubMed Central. There were also independent journals and other sources included. Other than databases, the literature was sourced from publications such as the Journal of Thrombosis and Thrombolysis, JAMA Network, BMJ, Elsevier, American Heart Association (AHA) Journal, and others.

Search Strategy

The search strategy was established based on the PICOS scheme (discussed later) and aimed at retrieving only the most relevant data from the digital databases. In the current search strategy, a total of 21 studies (out of a total sample of n =730) were eligible. We developed the PubMed search string and covered the following terms: (“atrial fibrillation” OR “AF”) AND (“rivaroxaban” AND (“stroke prevention” OR “efficacy” OR “safety”), Filters: Abstract, Free full text, Clinical Study, Clinical Trials, in the last 6 years, Humans, English.

Selection Process

Three researchers looked through peer-reviewed journals and publications for literature that met the inclusion criteria. Peer-reviewed journals with a high impact factor were investigated after a careful selection of the literature to lower the possibility of publication bias. For primary and secondary literature screening, all chosen studies were uploaded to Rayyan.ai, a screening program [10]. In order to “include” or “exclude” appropriate studies based on the inclusion and exclusion criteria, three researchers collaborated. For the final review and analysis, 21 studies (n = 1,291) were taken into account. Research that failed the eligibility requirements for screening was classified as “dispute” or “exclusion.” In order to act as tiebreakers for a disputed study, we assembled a team of three researchers for study selection. Studies that (1) had a different population, (2) had a design and methodology that was not appropriate for inclusion, (3) calculated incorrect outcomes, or (4) had a high risk of bias were excluded. Occasionally, there was a combined effect from several exclusionary factors.

Data Items

**Figure 1:**
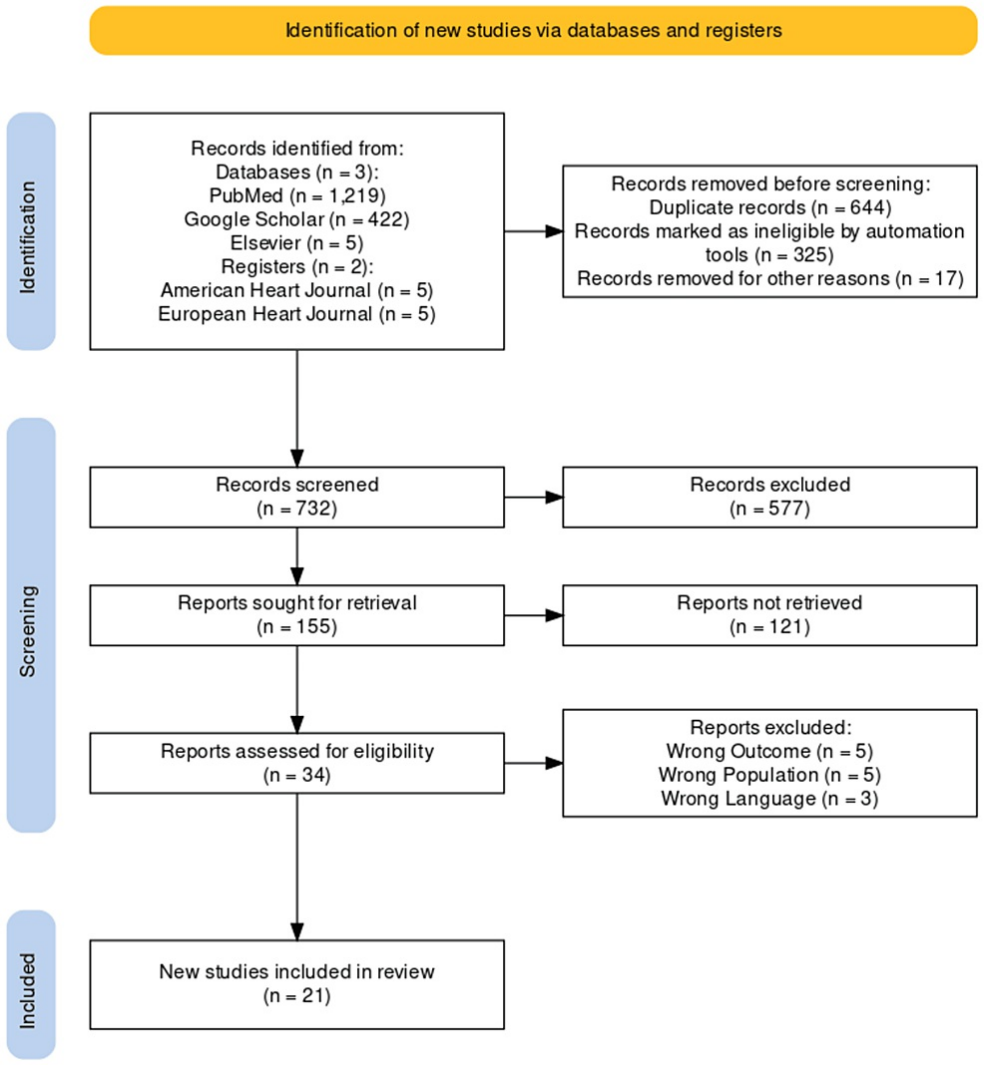
PRISMA flowchart for selected studies

Following the completion of the secondary screening protocol, the total sample size (n =21) for the chosen literature was evaluated. For the chosen studies from journals and other independent resources (if the reports were available), we created a PRISMA flow diagram using the PRISMA standards [11] (Figure 1). To reduce bias in the analysis, the following measures were taken: (1) choosing high-quality research, (2) requiring peer reviewers to disclose conflicts of interest, and (3) substituting meta-analyses for regular review articles. Systematic reviews and narrative reviews were excluded in order to maintain the study's standards. Following the stages of removing publication bias proposed by Chalmers et al., these guidelines identify and eliminate bias from the study protocol [12]. Based on these data, a "traffic light" figure was generated through randomization (Figure 1).

Assessment of Research Quality

Systematic review: Study bias was examined in all primary studies that were selected for assessment of quality. The demographics of the population, the studies' intervention features, and their outcome domains were all manually reviewed. All the studies selected for meta-analysis underwent quality assessment using the Critical Appraisal Skill Program (CASP) tool [13]. The quality assessment included three broad categories of questions: (1) Were the study results validated? (2) What were the results? (3) Are the results of the study applicable locally? 11 questions for quality assessment were answered with careful consideration of study designs and the relevant outcomes. The responses to the questions were "Yes," "No," and "Can't tell.” If the first question is answered in the affirmative, it makes logical sense to move on to the other inquiries. The questions overlap each other in certain ways. The description of the answers and researchers' remarks has also been mentioned in the assessment table (Table 2).

**Table 2:** Quality assessment using the Critical Appraisal Skill Program (CASP) tool Y = yes; N = no; ? = can’t tell CI, confidence interval; HR, hazard ratio

S. No.	Questions	Ray et al. [14]	Lau et al. [15]	Alberts et al. [16]	Huang et al. [17]	Jansson al. [18]
1	Was the study addressing a clearly defined problem?	?	Y	Y	Y	Y
2	Did the authors appropriately approach their research question?	Y	?	Y	Y	Y
3	Did the cases get recruited in a proper manner?	?	Y	Y	Y	Y
4	Were the controls chosen in a way that made logical sense?	Y	Y	Y	Y	Y
5	Was bias minimized by accurately measuring the exposure?	N	N	Y	Y	?
6(a)	Were the groups given the same treatment aside from the experimental intervention?	Y	Y	N	Y	Y
6(b)	Have the authors considered any potential confounding variables in their analysis or design?	N	Y	?	?	Y
7	How large was the treatment effect?	HR was 1.12, with a 95% CI of 1.05–1.21, and p=0.01	HR: 0.72; 95% CI: 0.66–0.79, p=0.01	The study predicted close HR values for outcome. HR: 0.81; 95% CI: 0.73–0.91, p=0.01	HR: 0.79; 95% CI: 0.66–0.94, p=0.01	The study showed a significant standardized absolute risk of 1.73% (1.43% to 2.03%
8	To what extent was the treatment effect estimate accurate?	p<0.0001. The results validate the study hypothesis	p=0.05. The overall effect size showed no significance	Analysis had a linear relation (p=0.05)	Statistically significant association, with p<0.001	Statistically significant association, with p<0.001
9	Do you think the outcomes are credible?	Y	Y	Y	Y	?
10	Can the local population employ the results?	Y	N	Y	N	N
11	Are the findings of this investigation consistent with other available data?	N	Y	?	Y	?
Score out of 11		7	9	9	10	8

Meta-analysis: In order to evaluate “bias” in the studies that were chosen, we looked for digital and online tools. With the exception of randomized controlled trials (RCTs), every study was evaluated using an online tool through CASP to produce a quality assessment table for every study that was a part of the meta-analysis (Table 2). Additionally, every primary study, that is, all RCTs that qualified for analysis, was chosen on its own using the Cochrane criteria for bias assessment [19]. The domains with potential for bias were as follows: (1) creation of a random sequence, (2) concealment of allocations, (3) blinding of participants and staff, (4) blinding of outcome assessments, (5) insufficient outcome data (attrition bias), (6) selective reporting (reporting bias), and (7) other biases. For the statistical meta, continuous data were taken from eight of the 21 primary studies. For the meta-analysis, we used Review Manager (RevMan version 5.4) to create a "forest plot." Rev-man (version 3.5.1) was used to conduct a meta-analysis of eight primary studies (study design = RCTs). For the analytical tool, three researchers gathered comparable and poolable data [20]. Every piece of information was accessible as continuous variables. The following section of our study contains the meta-analysis’s data.

Results

Study Characteristics

A total of 21 studies were manually selected in the final sample, of which 13 were RCTs and seven were cohort studies. Of these studies, 17 used randomization and 11 used a (quasi)-experimental design, of which eight used Cox regression methods to construct a matched comparison group. One study used latent curve modeling as well. The sample size of the populations in each study ranged from 175 to 227,572. Follow-up data ranged from 2 months to 24 months. The results of the systematic revealed a total of 19/21 (84%) studies advocating the effectiveness of rivaroxaban for stroke prevention. On the other hand, 2/21 (23%) studies concluded a “no effect” or “negative” association of rivaroxaban with the prevention of stroke. The comparative analysis of rivaroxaban versus warfarin also showed non-inferiority in the rivaroxaban-treated subjects. The synthesis table for the systematic review is given in Table 3.

**Table 3:** Results of the systematic review AF, atrial fibrillation; CAD, coronary artery disease; CrCl, creatinine clearance; DOACS, direct oral anticoagulants; DTI, direct thrombin inhibitors; FXaI, factor Xa inhibitors; HR, hazard ratio; ICH, intracranial hemorrhage; INR, international normalized ratio; NOACs, non-vitamin K antagonists; NVAF, non-valvular atrial fibrillation; ROCKET AF trial, Rivaroxaban Once-daily oral Direct Factor Xa Inhibition Compared with Vitamin K Antagonism for Prevention of Stroke and Embolism Trial in Atrial Fibrillation; TIA, transient ischemic attack; VKAs, vitamin K antagonists [14-18,21-36]

Sr. No.	Study	Location	Study Design	Participants	Intervention	Main Findings
1.1.	Ray et al. (2021) [14]	USA	Retrospective cohort study	581,451 patients from the US who were 65 years and younger and had disabilities.	Apixaban and rivaroxaban: standard (20 mg OD) or reduced (15 mg OD)	Compared to apixaban, rivaroxaban use showed a greater association with major ischemic or hemorrhagic events. Moreover, the frequency of non-cranial bleeding was higher with rivaroxaban.
2.	Lau et al. (2022) [15]	France, Germany, UK, USA	Multinational population-based cohort study	527,226 patients who started taking DOACs for treatment. These comprised 61,008 users of dabigatran, 12,722 users of edoxaban, 172,176 users of rivaroxaban, and 281,320 users of apixaban.	Apixaban, dabigatran, edoxaban, or rivaroxaban with standard as well as at reduced dose	DOACs lowered the risk of bleeding complications, such as gastrointestinal bleeding, and had a proportionate incidence of ICH, thromboembolism, and all-cause death in patients of AF.
3.	Alberts et al. (2020) [16]	USA	Retrospective cohort study	20,473 patients from the Optum Database have had their identities removed: warfarin (n = 13,597) and rivaroxaban (n = 6,876)	Patients who were treated with warfarin or rivaroxaban within 1 month of NVAF diagnosis	Individuals receiving rivaroxaban, as opposed to warfarin, experienced a notable decrease in the risk of stroke, as well as a reduction in “death due to all causes.”
4.	Huang et al. (2018) [17]	Taiwan	Retrospective cohort study	24,101 patients with an AF diagnosis who were 20 years of age or older satisfied the inclusion criteria (AF) between June 1, 2012, and December 31, 2015.	Rivaroxaban (20 mg, 15 mg, 10 mg) or warfarin therapy.	Rivaroxaban demonstrated a significantly lower risk for both composite effectiveness and safety outcomes when compared to warfarin.
5.	Jansson et al. (2023) [18]	Sweden	Registry-based retrospective cohort study	Out of a total of 40,561 patients, 29,481 were treated with warfarin, while 11,083 patients received treatment with recently initiated DOACs.	Patients receiving lower dosages of DOACs, such as rivaroxaban 15 mg OD, dabigatran 110 mg (BD), or apixaban 2.5 mg BD.	Differences in the rates of hemorrhagic stroke and all-cause stroke were significant, with HR values of 0.68 (0.50–0.92) and 0.87 (0.76–0.99), respectively. Compared to warfarin, rivaroxaban was associated with a higher risk of major bleeding, but it was also linked to better prevention of ischemic stroke.
6.	Carnicelli et al. (2022) [21]	Canada, Argentina, Taiwan, Scotland, Switzerland, USA, Germany, Japan	Randomized controlled trial	71,683 (29,362 on DOAC and 29,272 on warfarin).	In the ROCKET AF trial, the oral direct factor Xa inhibition trial (rivaroxaban once daily) was compared to VKA for the prevention of stroke and embolism.	Standard DOACs demonstrated a significantly decreased risk of stroke, mortality, and ICH when compared to warfarin. Conversely, there were no significant differences between low-dose DOACs and the risk of stroke.
7.	Guimarães et al. (2020) [22]	Brazil	Randomized controlled trial	1,005 adults, aged 18 and above, were prescribed oral anticoagulants to prevent thromboembolism, specifically for managing paroxysmal or persistent AF.	The study compared the effects of dose-adjusted warfarin, which aimed to achieve an INR between 2.0 and 3.0, with rivaroxaban at a daily dose of 20 mg.	When comparing the average time to the primary outcome—defined as death, major cardiovascular events, or major bleeding at the 12-month mark—rivaroxaban showed superiority to warfarin.
8.	Perera et al. (2020) [23]	Canada	Randomized controlled trial	Patients with a pre-existing thrombosis and other vascular diseases. Up until February 2017, randomization and follow-up were carried out with 27,395 participants.	Either rivaroxaban (5 mg BD), aspirin (100 mg OD), or rivaroxaban (2.5 mg BD) was prescribed to the participants.	In individuals with systemic atherosclerosis, the combination of rivaroxaban and aspirin had significant reductions in cardioembolic strokes.
9.	Blumer et al. (2021) [24]	Multi-national	Randomized controlled trial	1,878 patients (13.2%) from Latin America were taken into consideration in this subgroup analysis out of the 14,264 patients who were randomly assigned in the ROCKET AF trial.	Participants were selected randomly to receive either dose-adjusted warfarin or a fixed dose of rivaroxaban.	When comparing patients from different regions of the world, patients with AF showed comparable rates of bleeding, elevated extent of vessel-related mortality, and incidences of stroke and/or systemic embolism.
10.	Akao et al. (2021) [25]	Japan	A randomized, multicenter trial	AF and stable CAD patients, male and female, aged 20 years or older.	Two groups: those in the first group were given rivaroxaban (10 mg once daily) as the only treatment, while the second group received rivaroxaban plus an antiplatelet agent (a P2Y12 inhibitor or aspirin).	The efficacy indicators of rivaroxaban were significantly reduced, and there was no evidence of differing effects according to stroke risk. Furthermore, no statistically significant variation was seen between patient risk categories for any of the endpoints, which included heart failure, ischemic strokes, hemorrhagic strokes, and other cases of thromboembolism.
11.	Mehra et al. (2019) [26]	Germany, Singapore, UK, USA, Netherlands	Double-blind, randomized trial	Randomly, 2,507 patients received rivaroxaban and 2,515 received a placebo.	To investigate the safety and effectiveness of rivaroxaban in comparison to a test (placebo) in individuals with chronic heart failure.	Rivaroxaban lowered the risk for stroke or TIA when compared to a placebo.
12.	Healey et al. (2019) [27]	Canada, Rome, Australia, Mexico, USA, Japan, China, Spain	Randomized controlled trial	7,213 patients with non-embolic stroke of unknown source and had recently experienced an ischemic stroke were enrolled in the trial.	After random sampling, patients were given (1) 100 mg of aspirin and (2) 15 mg of rivaroxaban with a once-daily regimen.	When taken by patients who have significant left atrial enlargement and embolic stroke, rivaroxaban has been shown to reduce the risk of recurrent stroke.
13.	Zhang et al. (2020) [28]	45 countries	Multicenter, randomized, double-blind trial	Electrocardiography revealed 14,264 patients with NVAF who were at high risk for stroke.	Patients were randomized to receive dose-adjusted warfarin (target INR of 2.0–3.0) or rivaroxaban (20 mg once daily [OD], with a follow-up period of 707 days.	A history of stroke and CrCl was significantly associated with efficacious outcomes. For NVAF, rivaroxaban dosages of 15 mg and 20 mg once daily are advised.
14.	Shrestha et al. (2017) [29]	USA	Retrospective, observational cohort study	Patients with NVAF who were adults and had at least one DOAC pharmacy claim, enrollment for at least 12 months following the initial DOAC claim, and had a documented CrCl.	The prescribed amount for apixaban was 5 mg or 2.5 mg twice a day; the prescribed dosage for dabigatran was 150 mg twice a day for those with a CrCl; and the recommended dosage for rivaroxaban was 20 mg once a day for patients whose CrCl was more than 50 mL/min and 15 mg once a day for patients whose CrCl was less than 50 mL/min.	69 patients (17.8%) out of the 388 eligible patients were given the wrong dosages; rivaroxaban had the highest rate of incorrect dosing. It is underlined that in order to reduce bleeding related to DOAC therapy, clinical factors other than renal function must be taken into account. There was no significant variation in the risk of stroke.
15.	Staerk et al. (2017) [30]	Denmark	Retrospective cohort study	Patients were included if they had been previously diagnosed with AF and went on to fill their first DOAC prescription.	The following dosages were used: 150 mg of dabigatran standard dose (n = 7,078), 20 mg of rivaroxaban standard dose (n = 6,868), and 5 mg of apixaban standard dose (n = 7,203).	The risk of associated stroke or thromboembolism did not differ significantly between standard and lowered dosages of oral anticoagulants that are NOACs.
16.	Pisters et al. (2017) [31]	Netherland	Prospective, observational study	6,784 patients who provided written informed consent were started on rivaroxaban therapy.	Rivaroxaban prescribed in three different regimens: (1) 20 mg OD, (2) 15 mg OD, and (3) 10 mg OD	Patients receiving rivaroxaban in routine clinical practice showed low rates of major bleeding.
17.	Ntaios et al. (2020) [32]	Multi-national	Randomized controlled trial	7213 patients were enrolled in the NAVIGATE-ESUS trial and followed up for a median of 11 months.	In the study, the efficacy of 100 mg of aspirin once daily and 15 mg of rivaroxaban once daily were compared.	Unidentified embolic stroke and paroxysmal embolic strokes were prevalent among the participants.
18.	Nagao et al. (2018) [33]	Japan	Randomized controlled trial	Between April 2015 and January 2018, 200 patients with non-valvular AF were prescribed a once-daily dose of rivaroxaban.	The patients were categorized into two groups: interrupted or non-interrupted doses. Once-daily DOACs, such as rivaroxaban and edoxaban, were administered in one group, while the other group received twice-daily DOACs, specifically rivaroxaban.	The incidence of silent stroke was significantly higher in the intervention group as compared to the control group.
19.	Guimarães et al. (2021) [34]	Brazil	Randomized controlled trial	The eligible participants for the study were individuals aged 18 years or older, diagnosed with AF or flutter, and had a bioprosthetic mitral valve.	Randomization process was used to assign participants to receive dose-adjusted warfarin with the intended INR. The follow-up period of the study was 12 months.	Rivaroxaban was found to be non-inferior to warfarin in the primary per-protocol analysis of the journal manuscript. The rivaroxaban arm experienced 1.7 events/100 patient-years, while the warfarin-treated patients experienced 2.2 events/100 patient-years (p<0.001 for non-inferiority).
20.	Karthikeyan et al. (2020) [35]	Multi-National	Randomized controlled trial	Eligible participants for the study were individuals aged 18 years and older with echocardiographically confirmed current or past AF or atrial flutter.	A central internet-based randomization system was used to assign patients to receive either rivaroxaban or VKA. Any locally authorized VKAs were given to patients assigned to VKA, with dosage adjustments made to keep the INR between two and three.	The incidences of vascular death or myocardial infarction are examples of secondary efficacy outcomes. The duration of non-major bleeding that is not life-threatening or clinically significant is another secondary safety outcome.
21.	Providência et al. (2014) [36]	N/A	Meta-analysis	All phase III randomized controlled trials examining the safety and effectiveness of DOACs in comparison to warfarin in patients with AF were deemed eligible for inclusion.	A common comparator was used to estimate the relative risk between treatment A (DTI) and treatment B (warfarin) as well as between treatment C (FXaI) and B (warfarin).	DOACs were found to have a better effect on AF than warfarin on the risks for stroke, overall cardiovascular mortality, and intracranial bleeding.

**Figure 2:**
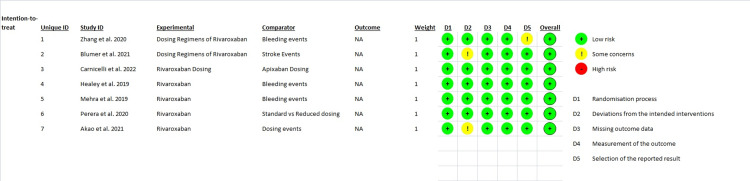
Cochrane ROB “traffic lights” plot 21,23-28

As was previously indicated, each study that was incorporated into the meta-analysis had its risk of bias evaluated. In the end, the final sample consisted only of the studies that demonstrated a “low” risk of bias across all domains. For the final evaluation, a “traffic lights” plot was made using the Cochrane ROBv2 tool. Figure 2 displays the ROB plot for seven primary studies.

Forest plots

Rivaroxaban Versus Warfarin 

**Figure 3:**
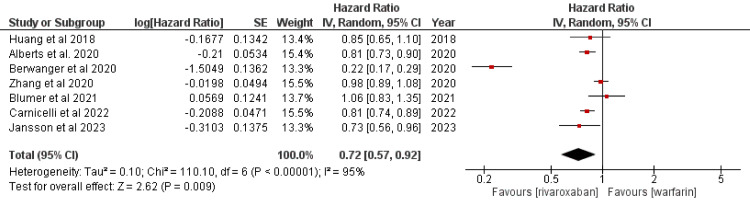
Forest plot for standard dose of rivaroxaban [16-18,21,22,24,28] CI, confidence interval

A forest plot was created using data from eight distinct studies, with the primary outcome being the generalized inverse variance measured by the hazard ratio (HR). To compute the hazard ratio (HR) in terms of “log[HR]” and standard error (SE), a random-effects model was selected. The horizontal axis estimated the confidence interval (CI=95%); the plot’s “point estimation” was presented as green squares. There was no significant change in the total sample size (n = 10,609, 175, 161, 2,060, 227,572, 29,362, 17,2176, 2,329) between the control groups. The vertical line in the center denotes a condition of “no effect.” The individual effects were found to be statistically significant for four out of seven studies: Alberts et al. [16], Huang et al. [17], Jansson et al. [18], and Carnicelli et al. [21]. The heterogeneity in the data was also investigated. It was given as follows: tau^2^=0.10; chi^2^=110.10, df=6; I^2^=95%. According to the analysis, Z=2.62 (p=0.009) represented the overall effect. The individual effect of all studies favored the rivaroxaban group. HR with a 95% CI was found to be 0.81 (0.73, 0.90) for Alberts et al. [16], 0.80 (0.65, 0.98) for Huang et al. [17], 0.86 (0.76, 1.06) for Jansson et al. [18], 0.81 (0.74, 0.89) for Carnicelli et al. [21], 1.06 (0.83, 1.35) for Blumer et al. [24], and 0.98 (0.89, 1.08) for Zhang et al. [28]. It indicates that individual effects of six out of eight studies favored the experimental group. According to this analysis, rivaroxaban performed better than warfarin in assisting patients with AF in reaching their treatment objectives. It was discovered that rivaroxaban was noticeably more effective in preventing stroke in the targeted patient group. Additionally, taking rivaroxaban increased the possibility of the primary outcome for both the reduced dose (RD) (6.4 [95% CI, 4.1-8.7]; HR= 1.28 [95% CI, 1.16-1.40]) and the standard dose (SD) (1.8 [95% CI, 1.0-2.6]; HR= 1.13 [95% CI, 1.06-1.21]). However, there was a significant difference in the SD group. The effect size was less significant when warfarin was compared with low-dose rivaroxaban strategies. This study conformed to the analysis laid down by another meta-analysis by Providência et al. (Figure 3) [36].

Incidence of Stroke After Rivaroxaban therapy

**Figure 4:**
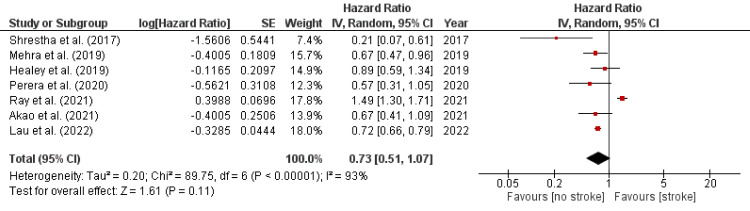
Forest plot for reduced dose of rivaroxaban [14,15,23,25-27,29] CI, confidence interval

The comparative efficacy of rivaroxaban was further investigated with the overall reduction in the incidence of stroke in patients treated with rivaroxaban. Further analysis also compared rivaroxaban with other DOACs. The risk of stroke in AF patients or valvular replacement was reduced in the patients treated with rivaroxaban. The combined effect for the analysis was found to be as follows: HR=0.73 (0.50, 1.07). The total effect was calculated to be Z=1.61 (p= 0.10). The heterogeneity was found to be as follows: tau^2^= 0.20, chi^2^=89.97, df=6, I^2^=93%. The individual effect sizes were found to be significant in six out of the following seven studies: Ray et al. [14], Lau et al. [15], Perera et al. [23], Akao et al. [25], Mehra et al. [26], Healey et al. [27], and Shrestha et al. [29]. The individual effect of all studies favored the “no stroke” group. HR with a 95% CI was found to be 1.49 (1.30, 1.70) for Ray et al. [14], 0.72 (0.66, 0.79) for Lau et al. [15], 0.57 (0.31, 1.03) for Perera et al. [23], 0.67 (0.41, 1.08) for Akao et al. [25], 0.67 (0.47, 0.95) for Mehra et al. [26], and 0.89 (0.59, 1.35) for Healey et al. [27]. The forest plot for RD of rivaroxaban is shown in Figure 4.

Discussion

The systematic review and meta-analysis aimed to provide a comprehensive evaluation of the available evidence regarding the diverse dosing strategies of rivaroxaban in the context of a-fib. AF, a frequently encountered cardiac disorder, poses a significant hazard of stroke, and anticoagulation therapy plays a pivotal role in its management. The effectiveness of rivaroxaban was measured based on its efficacy and safety. The efficacy is determined by the relative decrease in the “incidence of stroke” and reduction in overall risk and frequency of thromboembolic events that lead to stroke.

Similarly, the safety of rivaroxaban was indicated by the frequency of adverse events, such as intracranial bleeding, heart failure, and vascular compromise, and their progression into complications. The relative effectiveness was also demonstrated by comparing the therapeutic effect of rivaroxaban with that of aspirin, vitamin K antagonists, and warfarin. In a study conducted by Perera et al. [23], low-dose rivaroxaban when used in combination with aspirin in patients with atherosclerosis showed a more significant reduction in the incidence of thromboembolic events and stroke. The current study also elaborated that a reduced incidence of ischemic stroke, cardioembolic events, and all-cause mortality post-treatment are directly associated with the safety index of rivaroxaban. In a study conducted by Huang et al. [17], composite effectiveness outcomes (HR=0.79%, 95% CI=0.66, 0.94) and safety outcomes (HR=0.83, 95% CI=0.71, 0.97) had better scores in incidence as well as risk of stroke in patients who were administered rivaroxaban when compared to warfarin therapy.

According to Miyazaki et al., under-dosing in most cases leads to a higher risk of bleeding [37]. Furthermore, different dosing regimens of rivaroxaban were also considered within the current analysis. Standard dosing is defined as a 20 mg prescription to be taken with a once-daily schedule. Reduced dosing, on the other hand, is a 15 mg dose prescribed for twice-daily use. Dose adjustments are clinically safe for patients with renal function loss and reduced creatinine clearance. When compared to a reduced dosing, standard dosing regimens showed a lower risk of major bleeding events (HR=0.80, 95% CI=0.65, 0.99). A study by Patel et al. presented similar conclusions regarding the efficacy of standard-dose rivaroxaban [1]. In contrast to this, a meta-analysis by Lee et al. reported outcomes that yielded slightly different findings from our data [38]. The discrepancies in this data can be a result of different study designs, non-similar patient cohorts, and different inclusion criteria [39]. The studies that showed a negative association between studied outcomes were by Staerk et al. [30] and Providencia et al. [36]. Hylek et al. [40] also investigated the safety profiles of direct oral anticoagulants in patients with AF. The study established evidence for the need for customized anticoagulation strategies, specifically considering bleeding risks and patient compatibility [40].

In the studies by Graham et al. and Fralick et al., individuals were prescribed rivaroxaban doses (constituting 23% of the participants) with underlying health conditions, which suggested a heightened vulnerability to variations in the effectiveness and safety of anticoagulants. This also emphasizes the critical significance of selecting the appropriate anticoagulant in this specific population [41,42]. According to a study by Staerk et al., rivaroxaban showed a 1-year standardized absolute risk for major bleeding of 2.78% when SDs were compared, while dabigatran and apixaban showed lower absolute risk differences (−0.93% and −0.54%), respectively. Similar results were observed for major bleeding with reduced NOAC doses [31]. The previously discussed American studies by Graham et al., Hernandez and Zhang, Noseworthy et al., and another meta-analysis by Bai et al., also found an increased risk of bleeding associated with rivaroxaban compared with dabigatran [42-46]. In the study, conducted in Taiwan, Huang et al. evaluated rivaroxaban’s effectiveness in preventing ischemic stroke among Asians with non-valvular AF findings and demonstrated that in comparison to warfarin, rivaroxaban significantly reduced the risk of intracranial hemorrhage and venous thromboembolism [17]. The effectiveness of both the 20 mg and 15 mg doses was highlighted by the fact that they were linked to a lower risk of ischemic stroke. Nevertheless, there was no ischemic stroke risk reduction with the 10 mg dosage [28]. RD oral anticoagulants have shown clinically significant and favorable effectiveness and safety profiles compared to maintaining therapeutic range with warfarin treatment, as indicated by a recent study by Jansson et al. [18]. These are notably linked to a substantially reduced risk of intracranial bleeding. Treatment with RD direct oral anticoagulants is linked to a lower risk of major bleeding and all-cause stroke when compared to a cohort treated with warfarin. In particular, rivaroxaban therapy shows improved ischemic stroke prevention but carries a higher risk of significant bleeding.

In terms of efficacy, we observed a dose-dependent trend, whereby an increase in rivaroxaban dosage (from 10 to 15 mg and 20 mg) was associated with a notably decreased risk of ischemic stroke in contrast to warfarin. The 20 mg group showed the greatest reduction in risk, indicating that the standard dosage of 20 mg per day may be more appropriate, especially for patients who do not have a higher risk of bleeding. In contrast to the J-ROCKET AF and ROCKET AF studies, they found no significant difference in the risk of ischemic stroke between rivaroxaban and warfarin. In many studies, patients, as reported by Huang et al. [17], exhibited a lower baseline risk, potentially elucidating the heightened effectiveness of rivaroxaban. Additionally, the rivaroxaban group demonstrated a significantly reduced risk of venous thromboembolism, as Bauersachs et al. observed, highlighting its efficacy in preventing venous thromboembolism among patients with AF [47].

In terms of safety, we found that patients receiving rivaroxaban had a comparable risk of gastrointestinal bleeding and a noticeably lower risk of intracranial hemorrhage. Our data revealed an unexpected correlation between the 20 mg dose and an intriguing lowered risk of complications. On the other hand, the risk of intracranial hemorrhage was not considerably reduced by the 10 mg dosage. These results could be explained by differences between groups. Because clinical judgment was used by the physicians to determine the dosage in our study, patients in the 20 mg group may have been more robust and had a lower bleeding risk. In contrast, the 10 mg group included more fragile individuals with a higher bleeding risk, which may have contributed to the less noticeable reduction in intracranial hemorrhage risk [48]. Nonetheless, Gozzo et al. demonstrated a high frequency of prescriptions for low doses [48]. Dose adjustments, as mentioned, are considered clinically safe for patients with moderate to advanced-stage chronic kidney disease and reduced creatinine clearance [49]. The current study reaffirms that standard dosing of rivaroxaban consistently demonstrates superior efficacy in preventing composite effectiveness outcomes and ischemic strokes compared to reduced dosing. This has clear implications for AF patients with varying thromboembolic risk profiles. Clinicians may lean toward standard dosing, particularly in individuals with a higher risk of stroke, ensuring robust protection against ischemic events.

Clinicians and medical professionals often weigh the potential benefits and side effects before starting a patient on pharmacotherapy. Although standard dosing regimens are associated with certain side effects, the benefits and the impact on health outweigh the side effects. Some of the benefits include a reduced risk of major bleeding, gastrointestinal bleeding, and intracranial bleeding, which are known insults that significantly affect the quality-of-life indices of eligible patients. Consequently, there is a greater risk of bleeding with the RD regimens. The translation of evidence into practice ensures that AF patients receive anticoagulation therapy that is not only evidence-based but also reflective of real-world effectiveness and safety. Investigations into subpopulations, such as elderly patients or those with specific comorbidities, could provide personalized insights into the most effective and safe dosing strategies for these groups. Comprehensive, practical investigations are required to monitor the reliability of treatment and evaluate how well outcomes hold up in follow-up.

Strengths

The search strategy utilized in our study ensured the inclusion of a range of studies, providing a broad representation of the current literature on rivaroxaban dosing in AF. Rigorous inclusion criteria were applied, strengthening the quality of research and minimizing the risk of bias. Additionally, the use of a meta-analytic approach allowed for a quantitative synthesis of data, enabling assessment of the efficacy and safety outcomes associated with different dosing regimens. These methodological strengths collectively contribute to the robustness of the study’s conclusions, offering valuable insights into the optimal use of rivaroxaban in AF.

Limitations

Although the study investigated the right outcomes and measures for analysis and assessment, it had several limitations. First, the sample sizes taken for meta-analysis could not be standardized according to usual protocols. We used study characteristics in consideration but did not consider methodological characteristics of studies. Secondly, very few primary studies were utilized to assess the effectiveness (outcome domain) for such a large sample size. Thirdly, we evaluated the overall combined effect of all sample sizes, but within-group and sub-group analyses were not performed. Several studies have demonstrated that the results of the final analysis can be significantly altered when population demographics are sub-grouped into effect sizes.

## Conclusions

The clinical implications drawn from this analysis advocate for a thoughtful and patient-centered approach to anticoagulation therapy in AF. There is little impact of difference in the dosing of rivaroxaban with slightly reduced risk of stroke risk with standard dosing but with raised bleeding events. Moreover, our analysis moves beyond statistical significance to confirm the clinical significance of rivaroxaban’s effectiveness in protecting this patient population against stroke. By considering the individualized needs of patients, clinicians can navigate the complexities of stroke prevention and bleeding risk, optimizing outcomes in this high-risk population.
